# Phase 1 dose‐escalation study of single‐agent veliparib in Japanese patients with advanced solid tumors

**DOI:** 10.1111/cas.13307

**Published:** 2017-08-05

**Authors:** Tadaaki Nishikawa, Koji Matsumoto, Kenji Tamura, Hiroyuki Yoshida, Yuichi Imai, Aki Miyasaka, Takuma Onoe, Satoshi Yamaguchi, Chikako Shimizu, Kan Yonemori, Tatsunori Shimoi, Mayu Yunokawa, Hao Xiong, Silpa Nuthalapati, Hideyuki Hashiba, Tsukasa Kiriyama, Terri Leahy, Philip Komarnitsky, Keiichi Fujiwara

**Affiliations:** ^1^ Department of Gynecologic Oncology Saitama Medical University International Medical Center Saitama Japan; ^2^ Department of Breast and Medical Oncology National Cancer Center Hospital Tokyo Japan; ^3^ Department of Medical Oncology Hyogo Cancer Center Hyogo Japan; ^4^ Department of Gynecologic Oncology Hyogo Cancer Center Hyogo Japan; ^5^ AbbVie, Inc. North Chicago Illinois USA; ^6^ AbbVie GK Tokyo Japan

**Keywords:** High‐grade serous ovarian cancer, Japanese, phase 1, poly(ADP‐ribose) polymerase, veliparib

## Abstract

Veliparib (ABT‐888) is a potent, orally bioavailable poly(ADP‐ribose) polymerase‐1 and ‐2 inhibitor. This phase 1 study evaluated the tolerability, pharmacokinetic profile, safety, and preliminary antitumor activity of single‐agent veliparib in Japanese patients with advanced solid tumors. Eligible patients were assigned to treatment with veliparib at 200 or 400 mg dose; veliparib was self‐administered orally twice daily on days 1–28 of 28‐day cycles. Dose escalation, following a 3 + 3 design, defined dose‐limiting toxicities, the maximum tolerated dose, and the recommended phase 2 dose. Sixteen patients were enrolled (median age, 59 years). Fourteen patients had high‐grade serous ovarian cancer, one had primary peritoneal cancer, and one had *BRCA*‐mutated breast cancer. The most frequent treatment‐emergent adverse events were nausea and vomiting (93.8% each), decreased appetite (62.5%), abdominal pain, diarrhea, and malaise (31.3% each). A grade ≥3 toxicity was observed in 50% of patients; one patient each in the 200 mg (*n =* 4) and 400 mg (*n =* 12) cohorts experienced serious adverse events. Dose‐limiting toxicities were observed for one patient at the 400 mg dose. No toxicities leading to death were reported. The recommended phase 2 dose was defined as 400 mg twice daily. The veliparib pharmacokinetic profile was consistent with that reported for the Western population. Two patients, both with ovarian cancer, had a RECIST partial response. Veliparib monotherapy showed manageable tolerability and safety profiles and a predictable pharmacokinetic profile at a 400 mg twice‐daily dose, and supports the inclusion of Japanese patients in the multinational phase 3 study (NCT02470585).

Epithelial cancers of the ovary are the most common type of gynecologic malignancies, consisting of a heterogeneous group of histologically and genetically distinct diseases.^1^ While the mean age at diagnosis is 64 years,^2^ ~25% of cases are diagnosed between the ages of 35 and 54 years, with the highest rate in Caucasian women.^3^ Most cases of ovarian cancers (>70%) are diagnosed at an advanced stage. As a consequence, cancers of the ovary, fallopian tubes, and primary peritoneum are the seventh leading cause of cancer mortality globally and the fifth leading cause of cancer death in the United States.^4^ In Japan, the estimated incidence of ovarian cancer in 2015 was 10 400 (2.5% of all cancers in women), accounting for an estimated 4800 deaths (3.2% of all cancer deaths).^5^ Multiple histologic subtypes, including serous, mucinous, endometrioid, and clear cell carcinoma, have been described, and differences in the prevalence of the subtypes among racial and ethnic groups have been suggested. While serous ovarian carcinoma is the most common worldwide, representing nearly two‐thirds of cases of epithelial ovarian cancers in Western countries and 40.7% of cases in Japan, the clear cell carcinoma subtype is more frequent in Japan (27.6%) compared to Western countries (~10%).^6^

Approximately 10–15% of ovarian cancers are caused by hereditary gene mutations in the *BRCA1* or *BRCA2* genes^7^; the lifetime risk of developing ovarian cancer for women with *BRCA1* and *BRCA2* genetic mutations is 39% and 11–17%, respectively.^8^, ^9^ High‐grade serous ovarian cancer (HGSOC) is the most common subtype of epithelial ovarian cancer and has distinct molecular features, including a high frequency of inherited mutations in either *BRCA1* or *BRCA2* genes, p53 mutations, genetic instability, and epigenetic changes.^1^, ^10^ Approximately 90% of patients with HGSOC present with advanced‐stage disease, and this subtype accounts for 70–80% of deaths from ovarian cancer.^11^

The standard treatment for ovarian, fallopian tube, and primary peritoneal cancer involves surgery to remove all visible disease in the abdomen (surgical debulking), and chemotherapy. Despite initial positive response rates to platinum‐based chemotherapy, many patients develop resistance after the first or subsequent treatment cycles, and the incidence of disease recurrence is high.^12^ For these patients with a poor prognosis, there is a clear unmet medical need for novel strategies to improve survival.

In breast cancer, mutations in *BRCA1* or *BRCA2* genes account for ~5% of all breast cancers and 15–20% of all hereditary cancers.^9^, ^13^ Approximately 55–65% of women who inherit a *BRCA1* gene mutation and 45% of women who inherit a *BRCA2* gene mutation will develop breast cancer before the age of 70 years.^9^

In tumor cells that harbor *BRCA1 or BRCA2* mutations, the homologous recombination (HR) pathway that repairs damaged DNA is defective, resulting in a build‐up of chromosomal aberrations leading ultimately to cell death. These tumor cells are therefore highly sensitive to cytotoxicity caused by DNA‐damaging agents, such as platinum‐based chemotherapy. An exciting new class of anticancer drugs that exploit this sensitivity are the poly(ADP‐ribose) polymerase (PARP) inhibitors. PARPs are a family of 17 enzymes that catalyze the PARylation of proteins. PARP‐1 and PARP‐2 are essential in recognizing DNA damage, including single‐ or double‐strand DNA breaks,^14^ and facilitating DNA repair via mechanisms such as single‐strand break repair (SSBR), base excision repair, mismatch repair, nucleotide excision repair, and HR. Inhibition of PARP‐1 and PARP‐2 results in less‐efficient DNA repair; as a consequence, the cells are more susceptible to cytotoxicity induced by DNA‐damaging agents. Cancer cells carrying *BRCA* mutations are especially sensitive to PARP inhibition.^15^, ^16^, ^17^ This phenomenon, known as synthetic lethality, results in cell death in response to PARP inhibition even in the absence of other insults.^18^, ^19^ The PARP inhibitor olaparib has been approved by the European Medicines Agency^20^ and the FDA^21^ in patients with *BRCA*‐mutated ovarian cancer. Recently, two additional PARP inhibitors, rucaparib and niraparib, have also been approved by the FDA.^22^, ^23^ Since up to 50% of HGSOC is considered to have deficiencies in HR, this disease is particularly sensitive to PARP inhibition.^24^

Veliparib (ABT‐888) is a potent, orally bioavailable, selective inhibitor of PARP‐1 and PARP‐2, with K_i_s (inhibition constants) of 5.2 and 2.9 nM, respectively.^25^ A phase 1 study demonstrated antitumor activity of single‐agent veliparib in patients with *BRCA*‐mutated and *BRCA*‐wild type tumors, with an objective response rate (ORR) and clinical benefit rate of 40% and 68%, respectively, in *BRCA*‐mutated tumors.^26^ Single‐agent activity has also been demonstrated in a phase 2 study in *BRCA*‐associated recurrent epithelial ovarian, fallopian tube, or primary peritoneal cancer^27^ and in a phase 2 study of patients with *BRCA* germline mutation and platinum resistance, or partially platinum‐sensitive relapse of epithelial ovarian cancer.^28^

The primary objective of this phase 1 study was to assess the tolerability of veliparib as a single agent in Japanese patients with advanced solid tumors. The secondary objectives were to assess the pharmacokinetics (PK), safety, and preliminary activity of veliparib in this population.

## Materials and Methods

### Patients

Eligible patients were ≥20 years of age; had an Eastern Cooperative Oncology Group (ECOG) performance status of 0–1; had histologically or cytologically confirmed solid tumor diagnosis, and any of the following: recurrent HGSOC and had completed or discontinued platinum‐based therapy; *BRCA*‐mutated breast cancer and had received prior chemotherapy with anthracycline and/or taxanes; and advanced solid tumors with deleterious mutations of *BRCA*. In addition, eligible patients had a life expectancy of >12 weeks, and adequate renal, hepatic, and bone marrow function. Patients of child‐bearing potential agreed to use adequate contraception prior to study entry, for the duration of the study, and for up to 3 months following completion of therapy. Eligible patients were able to swallow and retain oral medicine, and had measurable or non‐measurable disease. Prior major surgery, radiation therapy, chemotherapy or hormone therapy, or treatment with investigational agents had to be completed within 4 weeks prior to study enrollment (exception: 6 weeks for mitomycin C and nitrosoureas); any anticancer Chinese medicine/herbal remedies had to be completed within 14 days prior to study enrollment. Patients with any clinically significant uncontrolled medical conditions or adverse events (AEs) from prior surgery or anticancer therapies that were not recovered to the National Cancer Institute Common Terminology Criteria for Adverse Events (NCI CTCAE) version 4.0^29^ grade ≤1 were excluded. Prior therapy with a PARP inhibitor was not allowed. Pregnant or lactating women were ineligible. In the expansion cohort, patients were excluded if they had received more than three lines of prior chemotherapy or had progressed at, or within, 6 months of completion of the last platinum‐based regimen (if the patient had never received a platinum‐based regimen, they were considered eligible if the regimen was not considered as standard of care for the cancer type). The study was approved by an independent ethics committee/institutional review board before initiation and was performed in accordance with the International Conference on Harmonisation guidelines, Good Clinical Practice guidelines, and the Declaration of Helsinki. All patients provided written informed consent prior to participation in the study. This study is registered with ClinicalTrials.gov (NCT02210663).

### Study design

This phase 1 study was an open‐label, multicenter, dose‐escalation study evaluating the tolerability and preliminary efficacy of veliparib as a single agent in Japanese patients with advanced solid tumors.

Eligible patients were assigned to one of two treatment groups (dose levels). Veliparib at a dose of 200 or 400 mg was self‐administered orally twice daily (BID) on days 1–28 of a 28‐day cycle. If the 400 mg BID dose was not tolerable, enrollment in an alternative veliparib 300 mg dose level was planned. Three to six patients were enrolled in each treatment group. Dose escalation was carried out according to a 3 + 3 design to define dose‐limiting toxicities (DLTs), and the maximum administered dose/recommended phase 2 dose (RP2D). A patient was considered eligible for DLT evaluation if they had completed cycle 1 of the assigned regimen with 80% compliance of veliparib. Any patients who prematurely discontinued cycle 1 or experienced an AE related to veliparib were assessed for DLT. DLTs were determined during the first cycle and were defined as: any grade 4 neutrophil count lasting >5 days; any grade 4 thrombocytopenia; febrile neutropenia; >4‐week delay starting next treatment cycle due to toxicity; an increase in creatinine to grade ≥3 that is not corrected to grade 1 or baseline within 24 h; grade ≥3 metabolic toxicities that are not corrected to grade ≤2 within 24 h (i.e., glucose changes or hypokalemia, hypomagnesemia, hyperuricemia, hypophosphatemia, and hyponatremia); any symptomatic grade 4 metabolic toxicities regardless of duration or ability to correct; and any other grade ≥3 non‐hematologic toxicities considered related to veliparib (excluding nausea, vomiting, diarrhea, and tumor pain that have not been treated effectively with medication). Patients received treatment for a maximum of 30 cycles or until treatment discontinuation. Following completion of cycle 1, subsequent cycles of therapy did not begin until the absolute neutrophil count was ≥1500 cells/mm^3^ and the platelet count was ≥100 000/mm^3^. Treatment could be postponed for up to 28 days due to toxicity; longer toxicity‐related delays would lead to study discontinuation.

The maximum tolerated dose (MTD) was defined as the highest dose level at which fewer than two of total six patients or <33% of patients (if the cohort was expanded beyond six patients) experienced a DLT. The RP2D was determined based on the observed DLTs and identified MTD; RP2D would not be dosed higher than the MTD. If the MTD was not reached, the RP2D was defined based on the safety and PK data. Prophylactic use of antiemetics and colony‐stimulating factors was not permitted during the DLT‐evaluation period, in order to clearly define the safety profile in Japanese patients.

The dose‐expansion cohort (including at least six patients) was enrolled at the RP2D to evaluate the durable tolerability of veliparib monotherapy. Prophylactic use of antiemetics was allowed in the dose‐expansion cohort.

### Safety, PK, and efficacy assessments

The safety of each treatment group was assessed by evaluating study drug exposure, AEs, serious AEs (SAEs), all deaths, changes in laboratory determinations, and vital signs parameters. AEs reported from the start of veliparib administration until 30 days after discontinuation of veliparib were collected. AEs were assessed according to the NCI CTCAE version 4.0.^29^ An independent data monitoring committee reviewed safety data for DLT evaluation.

PK data were analyzed from blood samples (3 mL) collected on day 1 of cycle 1 at 0 h (pre‐dose), and at 0.5, 1, 1.5, 2, 3, 4, 6, and 8 h after receiving the morning veliparib dose, and at 24 h post‐dose (before the veliparib dose on day 2 of cycle 1). Noncompartmental methods were used to determine the maximum observed plasma concentration (*C*_max_), the time to *C*_max_ (*T*_max_), the area under the plasma concentration‐time curve (AUC), and terminal elimination half‐life (*t*_1/2_).

To determine preliminary antitumor efficacy, baseline radiographic tumor assessments were conducted within 3 weeks prior to registration and thereafter every 8 weeks from cycle 1 day 1 and at the final visit, using the Response Evaluation Criteria In Solid Tumors (RECIST) version 1.1.^30^ In addition, the investigator evaluated the patient for evidence of clinical disease progression at each visit. Changes in measurable tumor lesions were assessed to determine the response rate, defined as proportion of patients with complete response (CR) or partial response (PR), and time to progression (TTP), defined as the number of days from the date the patient was dosed to the date the patient experienced an event of progressive disease (PD). All events of PD were included, regardless of whether the event occurred while the patient was taking veliparib or had previously discontinued. Changes from baseline in performance status were analyzed.

### Tumor marker

All enrolled patients with ovarian cancer or peritoneal cancer had measurements of cancer antigen 125 (CA‐125) on day 1 of cycle 1, every cycle (28 days) thereafter, and at the final visit. CA‐125 response was measured according to the Gynecologic Cancer InterGroup (GCIG) CA‐125 response definition.^31^

### Statistical analyses

Unless otherwise noted, for all statistical analyses, statistical significance was determined by a two‐sided *P‐*value ≤0.05. Descriptive statistics were used to describe baseline demographic variables, and summary statistics were computed for PK variables at each sampling time. PK variables were determined per patient and dose level. The safety of veliparib was assessed by evaluating veliparib exposure, AEs, SAEs, all deaths, and changes in laboratory determinations and vital sign parameters. The proportion of patients with CR or PR was estimated with a 95% confidence interval (CI) constructed for the estimated proportion. The distribution of TTP was estimated using Kaplan‐Meier methodology.

## Results

### Patient characteristics

A total of 16 female patients (four in 200 mg cohort, six in 400 mg cohort, and six in 400 mg dose‐expansion cohort) with a median age of 59 years (range, 43–83) were enrolled in this study and received at least one dose of veliparib. Fourteen patients had HGSOC, one patient had primary peritoneal cancer, and one patient had *BRCA*‐mutated breast cancer. All patients had received prior surgery and a median of three or more prior chemotherapies (range, 1–7). Demographics and baseline patient characteristics are summarized in Table 1.

**Table 1:** Baseline patient demographic and clinical characteristics (safety population)

Characteristic	Number (%)		
	Veliparib 200 mg BID (*n =* 4)	Veliparib 400 mg BID (*n =* 12)	Veliparib total (*n =* 16)
Age, years			
<65	3 (75.0)	8 (66.7)	11 (68.8)
≥65	1 (25.0)	4 (33.3)	5 (31.3)
Race			
Asian	4 (100)	12 (100)	16 (100)
Ethnicity			
Japanese	4 (100)	12 (100)	16 (100)
Median duration of disease, months (range)	49.8 (14.8–121.2)	53.1 (7.3–106.3)	53.1 (7.3–121.2)
ECOG PS			
0	2 (50.0)	11 (91.7)	13 (81.3)
1	2 (50.0)	1 (8.3)	3 (18.8)
Tumor burden at time of enrollment			
Locally advanced	2 (50.0)	0	2 (12.5)
Metastatic	2 (50.0)	12 (100)	14 (87.5)
History of oncologic surgery	4 (100)	12 (100)	16 (100)
History of radiation therapy	0	1 (8.3)	1 (6.2)
Primary tumor			
Ovarian	4 (100)	11 (91.7)	15 (93.8)
Breast	0	1 (8.3)	1 (6.3)
Peritoneal	0	1 (8.3)	1 (6.3)
Known *BRCA* mutation status			
*BRCA1* mutated	0	3 (25.0)	3 (18.8)
*BRCA2* mutated	0	0	0
Not mutated	0	1 (8.3)	1 (6.3)
Not tested	4 (100)	8 (66.7)	12 (75.0)
No. of involved sites at enrollment			
1	0	5 (41.7)	5 (31.3)
2	1 (25.0)	5 (41.7)	6 (37.5)
3	3 (75.0)	1 (8.3)	4 (25.0)
4	0	1 (8.3)	1 (6.3)
No. of prior regimens of cytotoxic therapy			
1	0	1 (8.3)	1 (6.3)
2	2 (50.0)	2 (16.7)	4 (25.0)
≥3	2 (50.0)	9 (75.0)	11 (68.8)
Platinum sensitivity†			
Platinum sensitive	2 (50.0)	9 (81.8)	11 (73.3)
Platinum resistant‡	2 (50.0)	2 (18.2)	4 (26.7)

†One patient with breast cancer in the 400 mg cohort was excluded. ‡“Platinum resistant” was defined as patients who progressed on or within 6 months of completion of the last platinum‐based regimen. BID, twice daily; *BRCA*, breast cancer susceptibility gene; ECOG PS, Eastern Cooperative Oncology Group performance status.

### Safety

All 16 patients had discontinued treatment due to disease progression (75%), AEs/toxicity (6%), or withdrawal of consent (19%). All patients received a median of 4.5 cycles of veliparib (range, 1–12) and a median of 133.5 days of veliparib (range, 10–338 days). Patients in the 400 mg cohort received a median of 165.0 days (range, 15–338 days).

One patient in the 400 mg BID veliparib cohort experienced a DLT of grade 3 nausea, vomiting, decreased appetite, and fatigue. All sixteen patients (100%) in the safety population experienced at least one grade ≥1 TEAE. TEAEs that occurred in 20% or more of patients are summarized in Table 2. The majority of AEs were mild to moderate in intensity (grades 1 or 2). The most frequently reported toxicities (all grades) were nausea (93.8%) and vomiting (93.8%), decreased appetite (62.5%), abdominal pain, diarrhea, malaise, and dysgeusia (31.3%, each), neutropenia, thrombocytopenia, fatigue, and insomnia (25%, each). Overall, nausea (93.8%, all grades) and vomiting (87.5%, all grades) were the most common TEAEs related to treatment with veliparib, experienced by three (75%) patients each in the 200 mg cohort, and by 12 (100%) and 11 (91.7%) patients, respectively, at the 400 mg dose level. A grade ≥3 AE was observed in 50% of patients, including two patients experiencing grade 3 nausea during veliparib 400 mg treatment; two patients experienced a SAE, one each during treatment with veliparib 200 mg and veliparib 400 mg. AEs leading to veliparib dose interruption, reduction, or delay were reported in one (25%) patient in the 200 mg dose cohort (pelvic infection), and in seven (58.3%) patients in the 400 mg dose cohort, including nausea, fatigue, neutropenia and thrombocytopenia (in three patients each), vomiting (in two patients), and anemia, decreased appetite, and essential tremor (in one patient each). There were no AEs or SAEs leading to death. The RP2D of veliparib monotherapy was established as 400 mg BID.

**Table 2:** TEAEs in ≥20% of patients (safety population)

TEAE, *n* (%)	Veliparib 200 mg BID (*n =* 4)		Veliparib 400 mg BID (*n =* 12)		Veliparib total (*n =* 16)	
	All grade	Grade 3/4	All grade	Grade 3/4	All grade	Grade 3/4
Any AE	4 (100)	2 (50.0)	12 (100)	6 (50.0)	16 (100)	8 (50.0)
Blood and lymphatic system disorders						
Neutropenia†	0	0	4 (33.3)	2 (16.7)	4 (25.0)	2 (12.5)
Thrombocytopenia‡	0	0	4 (33.3)	0	4 (25.0)	0
Gastrointestinal disorders						
Abdominal pain	3 (75.0)	0	2 (16.7)	0	5 (31.3)	0
Diarrhea	1 (25.0)	0	4 (33.3)	1 (8.3)	5 (31.3)	1 (6.3)
Nausea	3 (75.0)	0	12 (100)	2 (16.7)	15 (93.8)	2 (12.5)
Vomiting	4 (100)	1 (25.0)	11 (91.7)	1 (8.3)	15 (93.8)	2 (12.5)
General disorders						
Fatigue	0	0	4 (33.3)	1 (8.3)	4 (25.0)	1 (6.3)
Malaise	2 (50.0)	0	3 (25.0)	0	5 (31.3)	0
Metabolism and nutritional disorders						
Decreased appetite	1 (25.0)	0	9 (75.0)	1 (8.3)	10 (62.5)	1 (6.3)
Nervous system disorders						
Dysgeusia	0	0	5 (41.7)	0	5 (31.3)	0
Psychiatric disorders						
Insomnia	0	0	4 (33.3)	0	4 (25.0)	0

†Including neutrophil count decreased. ‡Including platelet count decreased. BID, twice daily; TEAE, treatment‐emergent adverse event.

### PK

**Figure 1:**
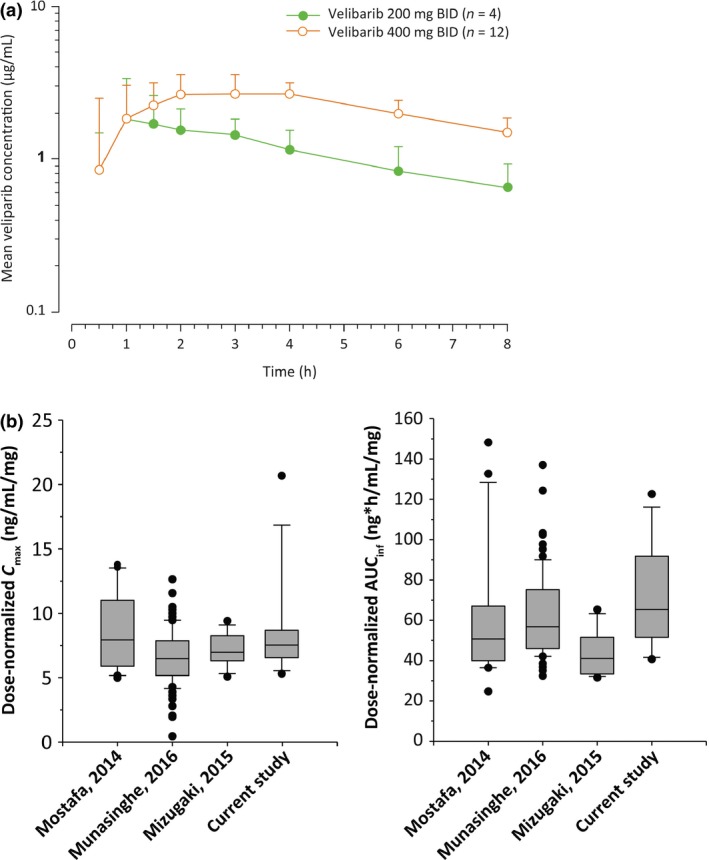
(a) Mean (+SD) plasma concentration‐time profile of veliparib on cycle 1 day 1 following oral administration of 200 and 400 mg veliparib. (b) Comparison of dose‐normalized PK parameters of veliparib across studies. AUC_inf_, area under the plasma concentration‐time curve from time 0 to infinity; BID, twice‐daily; *C*_max_, maximum plasma concentration; h, hours; PK, pharmacokinetic.

Following single‐dose oral administration of veliparib 200 mg and veliparib 400 mg, veliparib *C*_max_ and AUC increased in a dose‐proportional manner, suggesting a linear PK profile (Table 3, Fig. 1a). The dose‐normalized *C*_max_ and AUC_inf_ values were similar to those observed in previous studies in Japanese and non‐Japanese patients^32^, ^33^, ^34^ (Fig. 1b).

**Table 3:** PK parameters of veliparib on cycle 1 day 1

Veliparib dose (mg)	*n*	*T*_max_ (h)†	*C*_max_ (μg/mL)	AUC_0–8_ (μg·h/mL)	AUC_inf_ (μg·h/mL)	*t*_1/2_ (h)‡
200	4	2.5 (1.0–3.0)	2.06 ± 1.39	8.89 ± 3.77	13.6 ± 6.24	4.6 ± 1.1
400	12	2.5 (0.5–4.0)	3.28 ± 1.04	16.6 ± 3.59	29.5 ± 9.82§	4.6 ± 1.3§

Data presented as mean ± SD. †*T*_max_ presented as median (min–max). ‡Harmonic mean ± pseudo SD. §*n =* 9. AUC_inf_, area under the plasma concentration‐time curve from time 0 to infinity; AUC_0–8_, area under the plasma concentration‐time curve from hour 0 to 8; *C*_max_, maximum observed plasma concentration; h, hour; PK, pharmacokinetics; *t*_1/2_, terminal elimination half‐life; *T*_max_, time to *C*_max_.

### Efficacy

**Figure 2:**
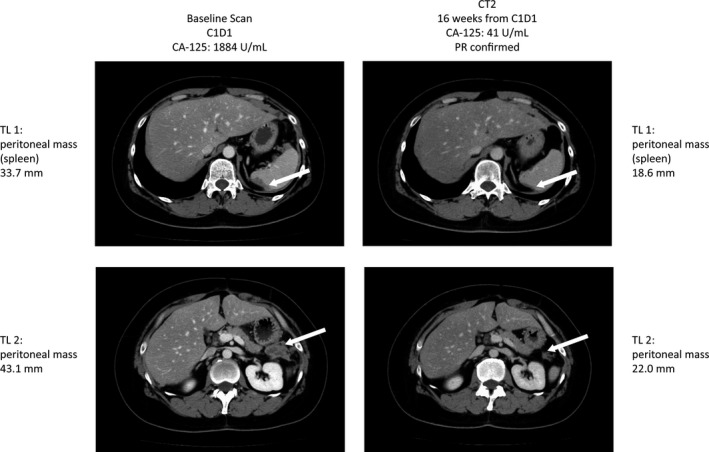
Computed tomography images showing partial response in a single patient to veliparib 200 mg BID. BID, twice‐daily; C1D1, day 1 of cycle 1; CA‐125, cancer antigen 125; CT, computed tomography; PR, partial response; TL, target lesion.

In total, 14 patients with measurable disease at baseline – 12 with ovarian cancer, one with primary peritoneal cancer, and one with breast cancer – were assessed for response (four in 200 mg cohort and 10 in 400 mg cohort). Among patients with ovarian and primary peritoneal cancer, the median number of prior platinum‐based therapies was three (range 1–5), and eleven had platinum sensitive ovarian cancer. The ORR to veliparib monotherapy was 14.3% (CI 1.8–42.8%). The best objective response was PR in two patients, both with platinum sensitive ovarian cancer, and stable disease in eight patients, including six patients with ovarian cancer, one with breast cancer, and one with primary peritoneal cancer; the latter two patients carried *BRCA*‐mutated tumors (Table 4). Computed tomography images of the patient with partial response to veliparib 200 mg are shown in Figure 2. The disease control rate (DCR, CR + PR + stable disease) among patients with measurable disease was 71.4% (10/14). Three patients (21.4%) progressed at first tumor assessment. The best objective response for the two patients without measurable disease at baseline was non‐CR/non‐PD. Overall, disease control was observed for 12 of 16 patients (75%).

**Table 4:** Best response rate in the efficacy population

Response, *n* (%)	Veliparib 200 mg BID (*n =* 4†)	Veliparib 400 mg BID (*n =* 10)	Veliparib total (*n =* 14)
ORR‡ [95% CI]	1§ (25.0) [0.6–80.6]	1¶ (10.0) [0.3–44.5]	2 (14.3) [1.8–42.8]
CR	0	0	0
PR	1 (25.0)	1 (10.0)	2 (14.3)
Stable disease	1 (25.0)	7 (70.0)	8 (57.1)
PD	1 (25.0)	2 (20.0)	3 (21.4)
Not evaluable	1 (25.0)	0	1 (7.1)
DCR (CR + PR + stable disease)	2 (50.0)	8 (80.0)	10 (71.4)

†One patient could not be evaluated. ‡ORRs confirmed by RECIST version 1.1^(30)^ and including patients with at least one measurable lesion at baseline. §Background information on the patient: platinum‐free interval >39 months; number of prior regimens = 4, number of metastatic sites = 2 (peritoneum, ascites). ¶Background information on the patient: platinum‐free interval = 10 months; number of prior regimens = 3, number of metastatic sites = 1 (lymph node). BID, twice daily; CI, confidence interval; CR, complete response; DCR, disease control rate; ORR, objective response rate; PD, progressive disease; PR, partial response; RECIST, Response Evaluation Criteria In Solid Tumors.

**Figure 3:**
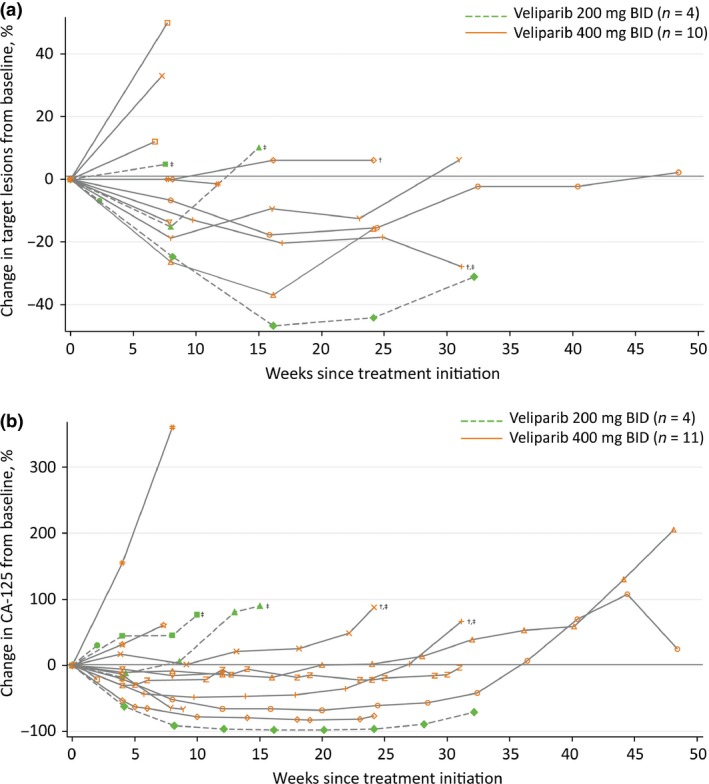
(a) Best percentage change from baseline in the sum of tumor sizes of target lesions (treated population, in patients with measurable disease at baseline). (b) Percentage change from baseline in CA‐125 (treated population^§^). CA‐125, cancer antigen 125. ^†^Patients with *BRCA* mutated tumor. ^‡^Platinum resistant patients – defined as patients who progressed on or within 6 months of completion of the last platinum‐based regimen. ^§^One patient with breast cancer was not included in the CA‐125 response analysis.

The percentage change from baseline in the sum of the tumor sizes of target lesions is shown in Figure 3(a). For all patients, the median percentage change from baseline was −10.3% (range, −46.8% to +50.0%).

Median TTP was 106 (range, 17–226) days, and 170 (range, 48–340) days for patients in the veliparib 200 and 400 mg dose cohorts, respectively. For patients with platinum sensitive ovarian or peritoneal cancer treated at 400 mg BID veliparib dose the median TTP was 218 days (range, 48–340).

### Tumor marker analysis

All patients with ovarian (*n =* 14) or primary peritoneal cancer (*n =* 1) had CA‐125 levels assessed. Three patients (20%) achieved CA‐125 response. One additional patient had a 50% reduction in CA‐125 levels, but this could not be confirmed 28 days from first assessment of response because of discontinuation from the study due to withdrawal of consent. Best percentage change from baseline values are presented in Figure 3(b). For all patients, the median change in CA‐125 level from baseline was −18.8% (18.8% reduction; range: −97.8% to +155.2%).

## Discussion

The results of this phase 1 study demonstrate that veliparib as monotherapy is well tolerated at the established RP2D of 400 mg BID in Japanese patients with advanced solid tumors. The most frequent AEs associated with single‐agent veliparib were nausea and vomiting, which is consistent with previous studies in Western patients,^26^, ^27^ and no new toxicities were identified. These toxicities may be manageable with antiemetics. The PK profile was consistent with previous Western studies^32^, ^33^ with a dose‐proportional PK profile, indicating that there are no differences in the PK of veliparib based on ethnicity. A recent phase 1 study of veliparib in combination with carboplatin/paclitaxel in Japanese non‐small cell lung cancer patients also showed a PK profile of veliparib comparable between Japanese and Western populations.^34^

Progress has been slow in improving outcomes for patients with ovarian cancer. Patients are often diagnosed at an advanced stage, and while initial responses to platinum‐based chemotherapy are good (>80%), the majority of patients eventually relapse and develop resistant disease.^12^ The goal of any treatment for these difficult‐to‐treat patients involves obtaining clinically meaningful objective responses while managing treatment toxicities to optimize quality of life.

Advances in the understanding of the molecular biology of epithelial ovarian cancer, and the impact of *BRCA1* and *BRCA2* gene mutations on cellular DNA repair pathways, has led to the development of PARP inhibitors, an exciting new class of targeted therapy. The exact mechanism of action of PARP inhibitors is an ongoing area of investigation; preclinical research suggests that PARP inhibitor activity may depend on different mechanisms of action. In the context of impaired HR resulting from *BRCA* mutations, PARP inhibitor cytotoxicity has been associated with the inhibition of SSBR, with the resulting accumulation of single‐ and double‐strand breaks causing replication fork collapse.^18^, ^19^ An additional mechanism of action whereby PARP is “trapped” on damaged DNA, resulting in cytotoxic PARP‐DNA complexes, has also been described recently.^35^

Antitumor activity was observed in two patients (14.3%), who had a PR to study treatment per RECIST version 1.1^30^ criteria, and three patients achieved CA‐125 response according to GCIG CA‐125 response definition. While the ORR was lower than that observed previously,^26^, ^27^ the DCR was 71.4% (10/14 patients), comparable to the Puhalla *et al*. (68%)^26^ and GOG‐280 studies (74.0%),^27^ where all patients carried a germline *BRCA1* or *BRCA2* mutation. Differences in the number of patients, the refractory nature of the disease, and *BRCA* status might explain the discrepancies in the results between studies.

Recent clinical trials of veliparib, both as monotherapy and in combination with a variety of chemotherapeutic agents or radiation therapy, have provided data in support of veliparib's activity in *BRCA‐*mutated tumors.^27^, ^36^, ^37^, ^38^, ^39^, ^40^, ^41^, ^42^, ^43^ In a phase 2 study of veliparib as monotherapy in patients with recurrent ovarian cancer and germline *BRCA1* and *BRCA2* mutations,^27^ 26% of patients showed an objective response (*n =* 50; 90% CI: 16–38%; CR: 2; PR: 11) with a median progression‐free survival of 8.2 months. For platinum‐resistant and platinum‐sensitive patients, objective responses were seen in 20% (*n =* 6/30; 90% CI: 9–36%) and 35% (*n =* 7/20; 90% CI:18–56%) of patients, respectively.^27^

The distinct limitation of this study for interpreting efficacy results is the small number of patients enrolled. Nevertheless, the sample size is sufficient to achieve study objectives – to evaluate PK variables and establish the RP2D of veliparib. Additionally, since a defective HR pathway is present in ~50% of HGSOC cases,^11^ and only three patients (18.8%) were confirmed as positive for *BRCA1* mutation in this study, this may have affected the efficacy results, given that PARP inhibitors are particularly effective in cells exhibiting BRCA1/2 deficiencies in the HR pathway for DNA repair. It should be noted that the median TTP in platinum sensitive ovarian cancer patients treated at RP2D appears comparable to that observed with olaparib and niraparib in the similar patient population.^16^, ^44^

In conclusion, this first study of veliparib as monotherapy in Japanese patients has demonstrated a manageable safety profile and confirmed that there are no differences in PK based on ethnicity, thereby supporting the inclusion of Japanese patients in the ongoing multinational phase 3 study (NCT02470585).

## Disclosure Statement

Koji Matsumoto has received honoraria from Chugai. Kenji Tamura has received research funding from Daiichi Sankyo, MSD, AstraZeneca, Eli Lilly, Pfizer. Satoshi Yamaguchi has received research funding from ZERIA Pharmaceutical. Chikako Shimizu has received research funding from Pfizer, Eli Lilly, Chugai. Kan Yonemori has received honoraria from Eisai. Hao Xiong, Silpa Nuthalapati, Terri Leahy and Philip Komarnitsky are employed by AbbVie and own AbbVie stock. Hideyuki Hashiba and Tsukasa Kiriyama are employed by AbbVie GK. Keiichi Fujiwara has received research funding from AbbVie, AstraZeneca, Pfizer, Chugai, Taiho, Eisai. Tadaaki Nishikawa, Hiroyuki Yoshida, Yuichi Imai, Aki Miyasaka, Takuma Onoe, Tatsunori Shimoi, Mayu Yunokawa have nothing to disclose.
